# Odor-specific olfactory stimulation is associated with selective transcriptional changes and partial improvement of stress-evoked bladder overactivity in mice

**DOI:** 10.3389/fnins.2026.1811702

**Published:** 2026-04-24

**Authors:** Saori Nishijima, Katsumi Kadekawa, Katsuhiro Ashitomi, Je Tae Woo, Hiroaki Kitano, Kimio Sugaya

**Affiliations:** 1 Southern Knights’ Laboratory, Okinawa, Japan; 2 Department of Biological Chemistry, College of Bioscience and Biotechnology, Chubu University, Kasugai, Aichi, Japan; 3 Integrated Open Systems Unit, Okinawa Institute of Science and Technology, Okinawa, Japan

**Keywords:** bladder overactivity, cystometry, essential oils, immediate-early genes, olfactory stimulation, stress-associated neural activation, transcriptomics, water avoidance stress

## Abstract

Chronic psychological stress is known to induce functional and molecular alterations in central circuits regulating micturition. Here, we investigated whether odor-specific olfactory stimulation modulates stress-associated bladder dysfunction and related brain transcriptional responses. Female C57BL/6J mice were subjected to repeated water avoidance stress (WAS) and exposed to inhalated essential oils derived from *Alpinia zerumbet* (Gettou) or *Citrus depressa* (Shikuwasa). Bladder function was assessed by continuous cystometry, spontaneous locomotor activity was evaluated during the dark phase, and whole-brain transcriptional responses were analyzed using exploratory RNA sequencing to characterize stress-associated transcriptional patterns. WAS was associated with bladder overactivity characterized by shortened contraction intervals, together with a tendency toward increased bladder weight and reduced locomotor activity. Transcriptomic analysis identified stress-associated upregulation of immediate-early genes and suppression of synapse-related gene categories. Gettou inhalation was associated with attenuation of stress-related immediate-early gene expression and partial normalization of bladder contraction dynamics, whereas Shikuwasa inhalation induced broad transcriptional changes without measurable improvement in bladder function. These findings suggest that odor-specific transcriptional modulation may be associated with partial functional improvement of stress-evoked bladder overactivity.

## Introduction

The brain plays a central role in integrating emotional stress and autonomic organ function, and lower urinary tract function is regulated by complex central and peripheral neural circuits (de Groat et al., 2015; Roy and Green, 2019). Chronic psychological stress is increasingly recognized as an important modifier of lower urinary tract function, influencing central neural mechanisms and altering multiple levels of the micturition pathway, thereby contributing to persistent bladder dysfunction (Gao and Rodríguez, 2022; Roy and Green, 2019; Shimizu et al., 2021).

In experimental models of stress-related bladder dysfunction, repeated exposure to water avoidance stress (WAS) reliably induces persistent visceral hypersensitivity (Bradesi et al., 2005). These functional alterations are accompanied by changes in sensory processing and neural regulation within the bladder pathway (Merrill et al., 2013; Vizzard, 2001).

A growing body of evidence suggests that sensory inputs, including olfactory stimuli, can modulate stress-related neural activity and autonomic balance (Herz, 2009; Sattayakhom et al., 2023). Given that stress-induced bladder dysfunction is mediated in part by central neural circuits, olfactory modulation of these circuits may represent a plausible mechanism for modulating micturition function. Aromatherapy has been reported to influence emotional regulation, hypothalamic–limbic activity, and stress responsiveness, raising the possibility that olfactory stimulation may affect physiological responses beyond emotional states alone (Herz, 2009; Sattayakhom et al., 2023). However, whether such neuromodulatory effects extend to stress-disrupted micturition pathways remains largely unexplored.

*Alpinia zerumbet* (Gettou) contains volatile monoterpenes that have been reported to exert anxiolytic-like effects following inhalation in rodents, and inhaled components have been detected in tissues including the brain (Murakami et al., 2009; Satou et al., 2010). *Citrus depressa* (Shikuwasa) essential oil contains monoterpenes such as limonene, which have been shown to exert anxiolytic and stress-modulating effects in rodents through serotonergic and related monoaminergic mechanisms (de Almeida et al., 2012; Komiya et al., 2006). Despite these reported neuromodulatory properties, the relative effects of different aromatic compounds on stress-evoked bladder dysfunction and central transcriptional responses remain unclear.

Accordingly, we hypothesized that odor-specific olfactory stimulation differentially modulates stress-evoked autonomic bladder dysfunction and associated brain transcriptional programs. By combining cystometric analysis with transcriptomic profiling, we aimed to characterize odor-specific modulation of stress-associated neural activation patterns linked to bladder overactivity.

## Materials and methods

### Animals and study design

Thirty-two female C57BL/6J mice (17.9–21.8 g, 8–10 weeks old) were housed under standard laboratory conditions (12-h light/dark cycle, lights on at 09:00, 22–24°C) with *ad libitum* access to food and water. Mice were randomly assigned to four groups: Control, Stress, Stress/Gettou, and Stress/Shikuwasa (*n* = 8 per group). For each group, six mice were used for physiological analyses (locomotor activity and cystometry), and a separate subset of two mice was used for transcriptomic analysis. All procedures were approved by the Okinawa Life Science Research Center and the Okinawa Institutional Animal Care and Use Committee (Approval No. SKL2025002).

### Water avoidance stress (WAS) and aroma inhalation

WAS was conducted according to established protocols (Smith et al., 2011). Mice were placed on a small platform (5.8 cm diameter × 8.5 cm height) in a cylindrical chamber (30 cm diameter × 28 cm height) filled with water such that the water level was 2 cm below the platform surface. For aroma exposure, a 2 × 2 cm filter paper containing 30 μL of essential oil (Alpinia zerumbet or Citrus depressa) was placed on the platform during each session. Essential oils were used as provided by the supplier (Okinawa Center for Research and Development of Natural Resources), stored according to the manufacturer’s instructions until use, and the same lot was used throughout the study. The chemical compositions of the essential oils used in this study were provided by the supplier and are summarized in Table 1. The major constituents of Gettou (Alpinia zerumbet) oil included α-pinene, camphene, p-cymene, limonene, and 1,8-cineole, whereas Shikuwasa (Citrus depressa) oil was dominated by limonene together with γ-terpinene and related monoterpenes. Stress-only mice were exposed to WAS with an untreated filter paper. Sessions were performed for 1 h/day between 19:00 and 21:00 for 8 consecutive days. This period corresponds to the active (dark) phase of mice, to minimize potential circadian influences on stress responses.

**TABLE 1:** Major chemical constituents of Gettou (*Alpinia zerumbet*) and Shikuwasa (*Citrus depressa*) essential oils.

Compound	Gettou (%)	Shikuwasa (%)
α-Pinene	7.80	2.9
β-Pinene	2.22	2.9
Myrcene	3.13	1.5
Limonene	8.25	73.9
y-Terpinene	0.49	31.1
p-Cymene	10.71	10.9
Camphene	9.21	1.1
Carene	0.22	1.7
Linalool	3.93	−
1,8-Cineole	8.14	−
Others	Remaining	2.4

Values represent relative percentages based on GC–MS peak areas provided by the supplier.

### RNA sequencing and transcriptomic analysis

Whole-brain RNA sequencing was conducted as an exploratory analysis to identify transcriptional patterns associated with stress and aroma exposure. Whole brains excluding the cerebellum were collected from two mice per group under deep isoflurane anesthesia and immediately processed for RNA extraction.

Total RNA was extracted using the RNeasy Mini Kit (Qiagen). RNA integrity was assessed using an Agilent Bioanalyzer prior to library preparation, and all samples used for RNA-seq showed RNA integrity numbers (RIN) ≥ 8. Poly(A)-selected library preparation and sequencing were performed by a commercial provider (KOTAI Biotechnology, Osaka, Japan) using paired-end 150 bp sequencing, generating approximately 40 million reads per sample. Quality control, alignment to the mouse reference genome (Ensembl), and gene-level quantification were performed by the service provider using standard RNA-seq pipelines. Downstream analyses included principal component analysis (PCA), hierarchical clustering, and differential expression analysis with false discovery rate (FDR) correction using the Benjamini–Hochberg method. Gene ontology enrichment analysis was conducted using over-representation methods.

To enhance transparency, differential expression results were further examined using iDEP with Benjamini–Hochberg correction. Genes with an adjusted *p*-value (FDR) < 0.05 were considered statistically significant. Given the limited biological replicates (*n* = 2 per group), transcriptomic results were interpreted cautiously, with emphasis placed on pathway-level trends and consistent transcriptional patterns rather than individual gene-level effects. RNA-seq data are publicly available under BioProject accession PRJNA1381277.

### Locomotor activity

Spontaneous locomotor activity was recorded using an infrared digital monitoring system (NS-ASS01; Neuroscience Inc., Tokyo, Japan). Activity was continuously measured during the dark phase (21:00–09:00) following the final WAS session and quantified as total activity counts over the recording period.

### Continuous cystometry

After locomotor recording, mice were anesthetized with urethane (total dose 0.6 g/kg; 0.2 g/kg i.p. and 0.4 g/kg s.c.). This relatively low dose was used to minimize potential effects of anesthesia on brain activity while providing sufficient sedation during cystometric recording. During the experiment, mice were gently secured to a cork board using adhesive tape to minimize movement. A PE-10 catheter was inserted transurethrally into the bladder and connected to a pressure transducer and infusion pump. Saline was infused at 1 mL/h. After stabilization for 60 min, at least four rhythmic micturition cycles were recorded.

### Statistical analysis

Group comparisons were performed using one-way analysis of variance (ANOVA) followed by Dunnett’s multiple comparison test, which compares each experimental group with the Control group while accounting for multiple comparisons. A *p*-value < 0.05 was considered statistically significant.

## Results

### Transcriptomic effects of stress

**FIGURE 1:**
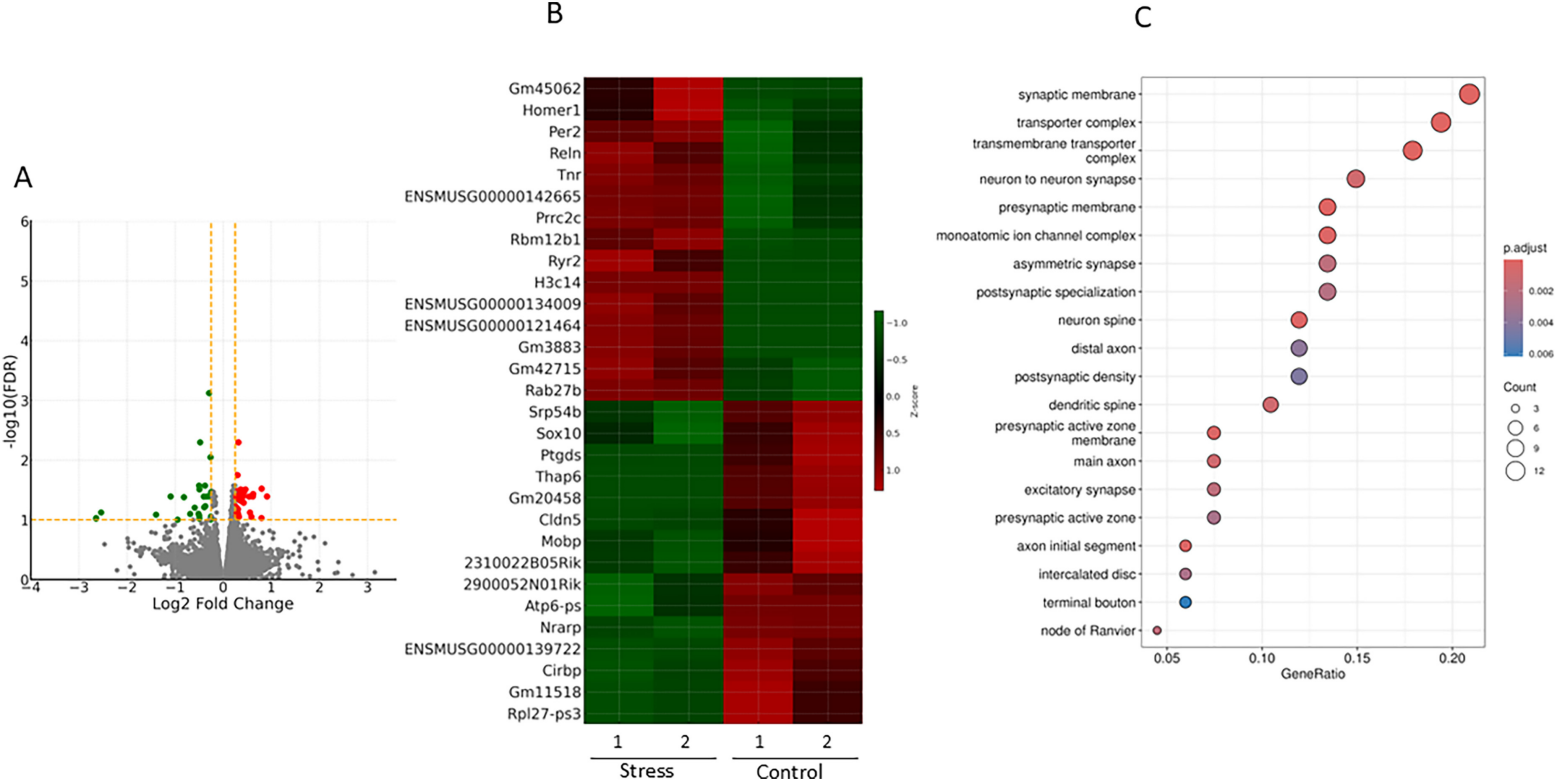
Transcriptomic changes associated with chronic stress (Control vs. Stress). Volcano plot showing differentially expressed genes between Control and Stress groups. Red dots represent upregulated genes and green dots represent downregulated genes (FDR < 0.05, | log_2_ fold change| ≥ 1) **(A)**. Heatmap of the top differentially expressed genes (Z-score normalized). Stress exposure was associated with altered expression of genes related to synaptic organization (e.g., *Homer1, Per2, Reln, Tnr*) **(B)**. Gene ontology (GO) cellular component enrichment analysis (bubble plot). GO analysis identified enrichment of categories related to synaptic structures, including synaptic membrane, postsynaptic density, and dendritic spines **(C)**. RNA-seq analysis was performed using *n* = 2 biological replicates per group and should therefore be interpreted as exploratory due to the limited sample size.

Stress exposure was associated with broad transcriptional alterations across the brain (excluding the cerebellum) (Figure 1A). Unsupervised heatmap clustering demonstrated separation between the Control and Stress groups (Figure 1B), suggesting stress-related shifts in global transcriptional patterns. Gene ontology (GO) analysis identified enrichment of synapse-related cellular components, including synaptic membrane, transporter complexes, neuron-to-neuron synapses, postsynaptic structures, dendritic spines, and distal axons (Figure 1C). Several genes related to synaptic organization and neuronal function, including *Per2, Homer1, Reln, Tnr, Cldn5*, and *Mobp*, showed reduced expression in stressed mice. These changes may reflect potential alterations in neural signaling and synaptic regulation.

### Effects of Gettou

**FIGURE 2:**
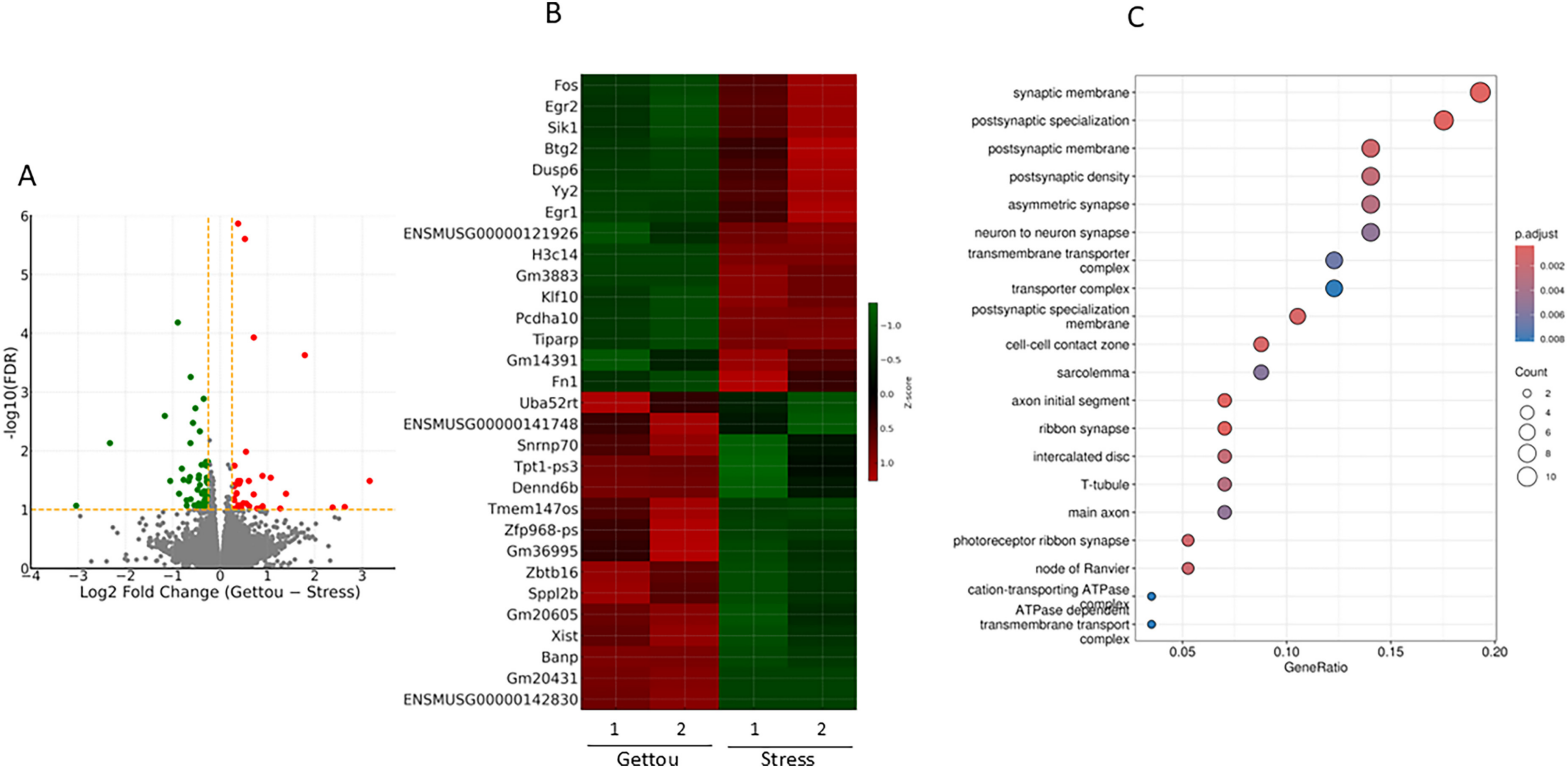
Transcriptomic changes associated with Gettou inhalation in stressed mice (Stress vs. Stress/Gettou). Volcano plot comparing Stress and Stress/Gettou groups **(A)**. Heatmap illustrating genes responsive to Gettou inhalation. Gettou inhalation was associated with reduced expression of several immediate-early genes (*Fos, Egr1, Sik1*, and *Dusp6*) relative to the Stress group **(B)**. GO cellular component enrichment analysis. Gettou inhalation was associated with modulation of categories related to synaptic structures, including postsynaptic density and synaptic membrane **(C)**. RNA-seq analysis was performed using *n* = 2 biological replicates per group and should therefore be interpreted as exploratory due to the limited sample size.

Gettou inhalation was associated with reduced expression of several activity-dependent immediate-early genes (IEGs), including *Fos, Egr1, Dusp6*, and *Sik1*, compared with the Stress group (Figures 2A,B). The overall transcriptional profile showed partial convergence toward that observed in control mice. GO enrichment analysis indicated enrichment of categories related to synaptic organization and intracellular signaling (Figures 2C).

### Effects of Shikuwasa

**FIGURE 3:**
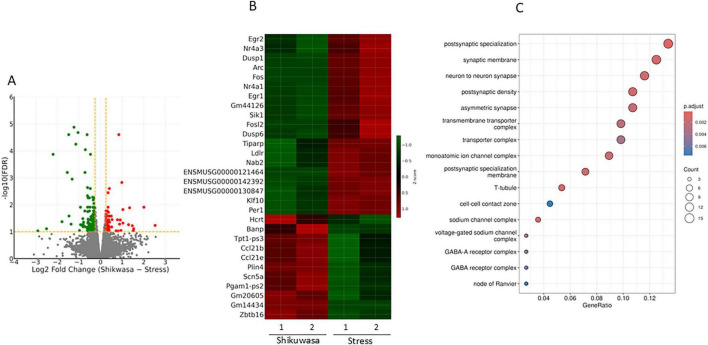
Transcriptomic effects of Shikuwasa inhalation in stressed mice (Stress vs. Stress/Shikuwasa). Volcano plot comparing Stress and Stress/Shikuwasa groups **(A)**. Shikuwasa inhalation was associated with both upregulated and downregulated genes relative to the Stress group. Heatmap of genes responsive to Shikuwasa inhalation **(B)**. Several immediate-early genes (*Fos, Egr1, Egr2, Arc, Nr4a1, Nr4a3, Sik1, Dusp6*) were lower in the Stress/Shikuwasa group compared with the Stress group, whereas a subset of transcripts (e.g., *Hcrt*) showed increased expression. GO cellular component enrichment analysis **(C)**. Shikuwasa inhalation was associated with enrichment of synapse- and ion channel-related categories. RNA-seq analysis was performed using *n* = 2 biological replicates per group and should therefore be interpreted as exploratory due to the limited sample size.

Shikuwasa inhalation was associated with broad transcriptional changes involving both up- and downregulated genes (Figures 3A,B). While expression of some immediate-early genes was reduced relative to the Stress group, other transcripts, including *Hcrt*, were increased. GO enrichment analysis identified enrichment of synapse- and ion channel-related categories, including postsynaptic specialization, synaptic membrane, sodium channel complexes, and GABA receptor complexes (Figures 3C), indicating modulation of synapse- and ion channel-related pathways. These transcriptional changes were not associated with apparent normalization of bladder contraction dynamics.

### Bladder function and organ weights

**FIGURE 4:**
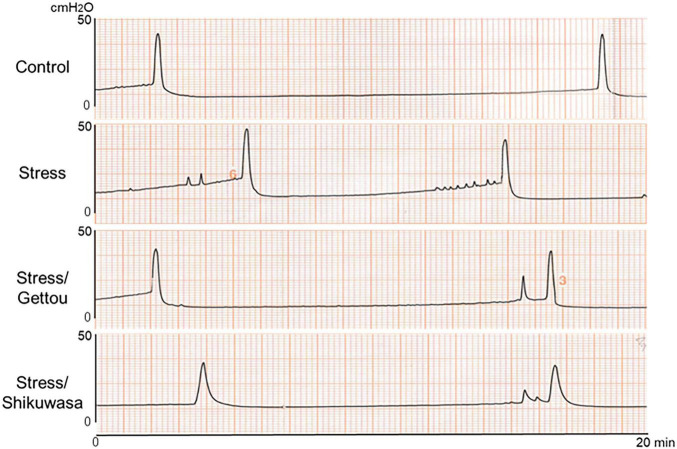
Representative cystometrogram traces from each experimental group (Control, Stress, Stress/Gettou, and Stress/Shikuwasa). The traces illustrate differences in bladder contraction intervals and baseline pressure among groups.

**FIGURE 5:**
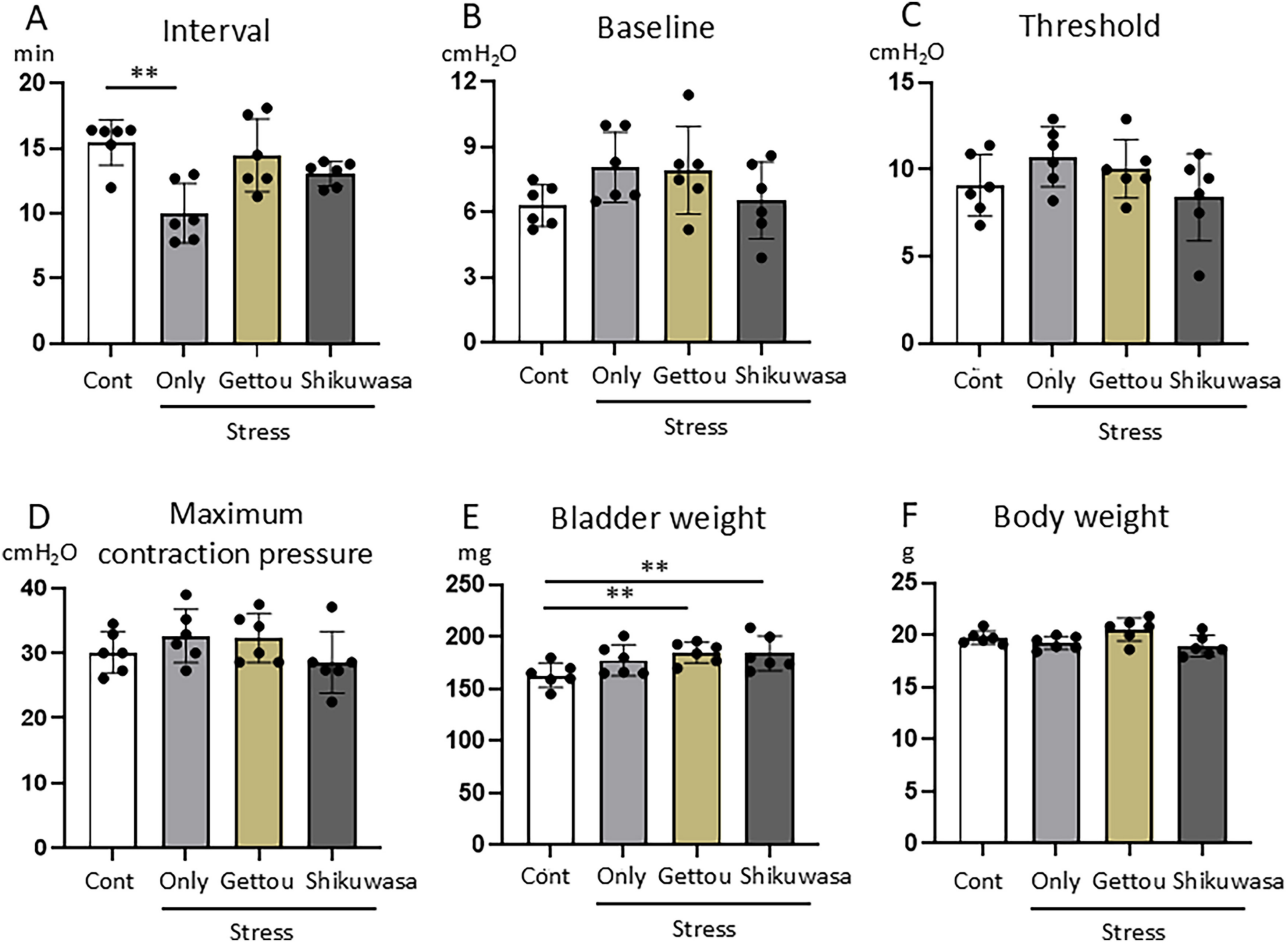
Effects of WAS and aroma inhalation on cystometric parameters, bladder weight, and body weight. Stress significantly shortened bladder contraction interval **(A)** compared with the control group. Gettou inhalation was associated with partial normalization of this parameter, whereas Shikuwasa inhalation did not restore the shortened contraction interval. Baseline pressure, threshold pressure, and maximum contraction pressure did not differ significantly among groups **(B–D)**. Bladder weight was significantly increased in the Stress/Gettou and Stress/Shikuwasa groups compared with the Control group, while the Stress group showed a non-significant increasing trend **(E)**. No significant differences in body weight were observed among groups **(F)**. Data are presented as mean ± SEM (*n* = 6 mice per group). Statistical analysis was performed using one-way ANOVA followed by Dunnett’s multiple comparison test. **p < 0.01 vs. control.

Water avoidance stress (WAS) significantly shortened the bladder contraction interval compared with controls (Figures 4, 5A). Gettou inhalation shifted bladder contraction interval toward control levels, whereas Shikuwasa inhalation did not restore the shortened contraction interval. Baseline pressure, threshold pressure, and maximum contraction pressure did not differ significantly among groups (Figures 5B–D). Bladder weight was increased in Stress/Gettou and Stress/Shikuwasa groups, while the Stress group showed a non-significant increasing trend (Figure 5E). Body weight remained unchanged (Figure 5F).

### Spontaneous locomotor activity

**FIGURE 6:**
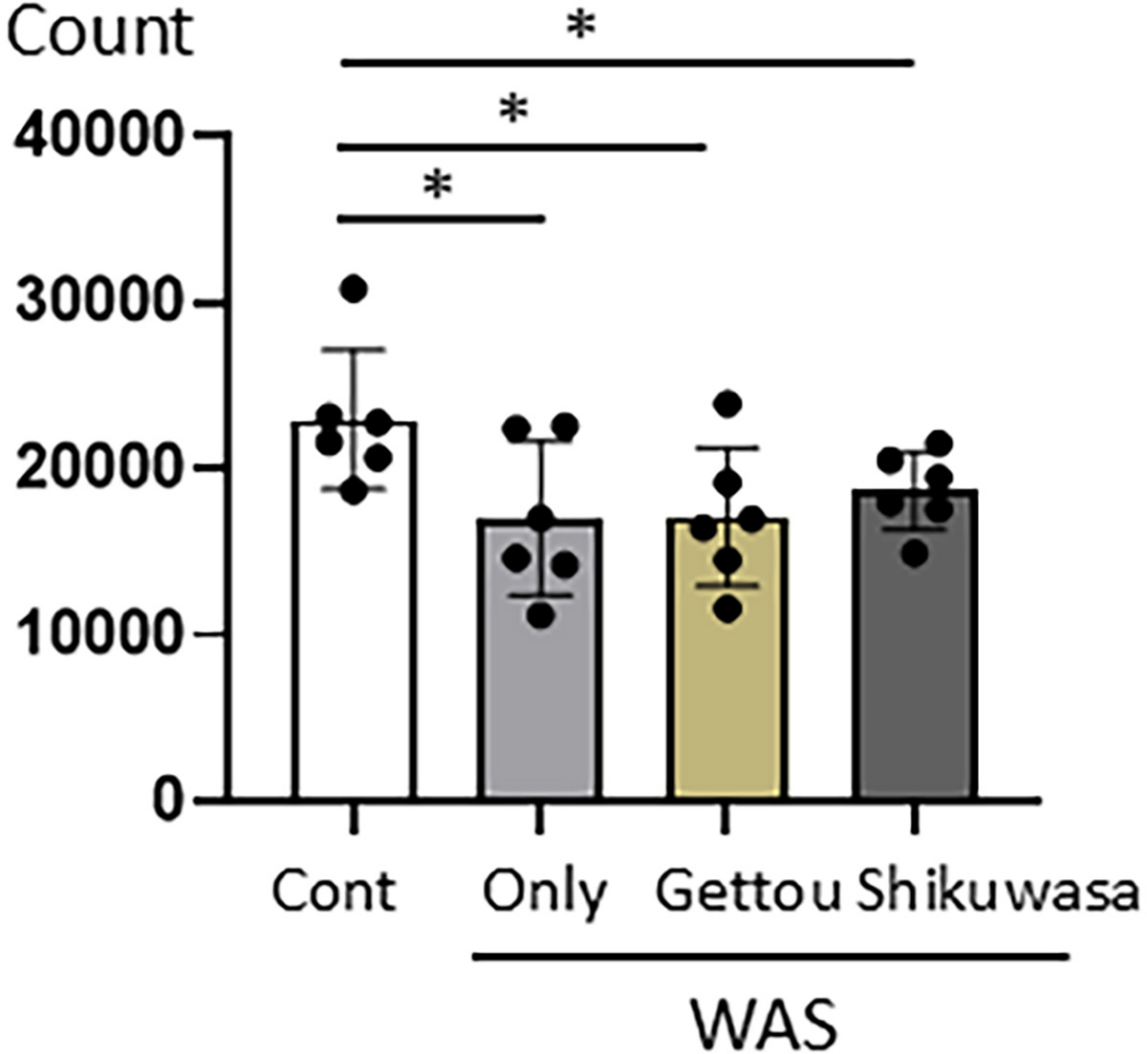
Spontaneous locomotor activity in control and WAS-exposed mice with or without aroma inhalation. Chronic stress significantly reduced spontaneous locomotor activity compared with the Control group. Neither Gettou nor Shikuwasa inhalation significantly altered the stress-associated reduction in activity. Data are presented as mean ± SEM (*n* = 6 mice per group). Statistical analysis was performed using one-way ANOVA followed by Dunnett’s multiple comparison test. *p < 0.05 vs. control.

All stress-exposed groups exhibited reduced spontaneous locomotor activity compared with the Control group (Figure 6). No significant differences were detected among the Stress, Stress/Gettou, and Stress/Shikuwasa groups, suggesting that aroma inhalation was not associated with a significant reversal of the stress-associated reduction in locomotor activity.

## Discussion

These findings suggest that olfactory input may modulate neural activation patterns associated with autonomic control of micturition. Chronic psychological stress was accompanied by coordinated changes in brain transcriptional activity together with functional alterations in bladder dynamics, indicating a potential link between stress-associated neural activity and bladder overactivity.

Repeated water avoidance stress (WAS) induced characteristic transcriptional alterations in the brain, including increased expression of immediate-early genes and reduced representation of synapse-related categories, suggesting stress-associated neural activation accompanied by alterations in synaptic organization. These neural changes occurred together with bladder overactivity characterized by shortened contraction intervals, a phenotype widely reported in experimental models of stress-related bladder dysfunction (Smith et al., 2011). Circadian mechanisms may also contribute to these alterations, as stress can influence neural regulatory processes partly through changes in clock gene expression, including *Per2* (Hood and Amir, 2017). Together with previous studies describing alterations in bladder afferent signaling (Vizzard, 2001) and disruption of central micturition circuits involving limbic, hypothalamic, and pontine regions (de Groat et al., 2015; Roy and Green, 2019), these findings suggest that stress-associated bladder dysfunction involves coordinated changes in both central neural regulation and peripheral bladder responses. Consistent with this interpretation, bladder weight also showed an increasing trend following stress exposure, in line with structural bladder alterations reported in stress models (Smith et al., 2011; Merrill et al., 2013).

Gettou inhalation was associated with attenuation of stress-related neural activation patterns. Expression of activity-dependent immediate-early genes such as *Fos* and *Egr* family members was reduced relative to the Stress group, suggesting an association with reduced neuronal activation within stress-responsive signaling pathways. Gene ontology analysis further indicated modulation of synapse-related structural categories. These transcriptional changes were accompanied by partial normalization of bladder contraction dynamics, raising the possibility that odor-specific modulation of neural activity may influence neural activity related to micturition control. The parallel attenuation of immediate-early gene expression and partial normalization of contraction intervals in the Gettou group further supports the idea that reduced stress-associated neural activation may contribute to functional improvement, although causal relationships cannot be established from the present data.

In contrast, Shikuwasa inhalation produced broader transcriptional remodeling involving both up- and downregulated genes. Although several immediate-early genes were reduced relative to the Stress group, other transcripts, including Hcrt, were increased, and diverse synapse- and ion channel-related pathways were affected. Despite these transcriptional changes, bladder contraction dynamics were not restored. Moreover, neither aroma reversed stress-associated reductions in locomotor activity or increases in bladder weight. Chronic stress is known to induce persistent neuroendocrine and inflammatory alterations that may not be readily reversed by short-term sensory modulation (Gao and Rodríguez, 2022; Merrill et al., 2013). These observations suggest that olfactory input may preferentially influence specific neural regulatory circuits rather than globally reversing stress-associated adaptations.

The transcriptomic analysis in this study was exploratory and based on a limited number of biological replicates. Therefore, the observed transcriptional changes should be interpreted as system-level associations rather than direct causal mechanisms. In addition, because whole-brain tissue excluding the cerebellum was analyzed, region-specific transcriptional changes within defined micturition-related nuclei could not be resolved. Future studies focusing on specific brain regions, such as the periaqueductal gray, pontine micturition center, hypothalamus, and limbic structures, will be necessary to clarify the neural pathways involved.

Overall, the present findings suggest that sensory inputs may influence stress-associated neural activity patterns and thereby contribute to changes in autonomic organ function. The aroma-specific differences observed here highlight the potential importance of selective neural stabilization, rather than widespread transcriptional remodeling, in the regulation of stress-associated micturition changes.

## Data Availability

The datasets presented in this study can be found in online repositories. The names of the repository/repositories and accession number(s) can be found in the article/supplementary material.
